# AI-Generated Face Image Identification with Different Color Space Channel Combinations

**DOI:** 10.3390/s22218228

**Published:** 2022-10-27

**Authors:** Songwen Mo, Pei Lu, Xiaoyong Liu

**Affiliations:** 1 Information Science and Engineering, Guilin University of Technology, Guilin 541004, China; 2 Guangxi Key Laboratory of Embedded Technology and Intelligent System, Guilin University of Technology, Guilin 541004, China

**Keywords:** deepfake, color space, attention mechanisms, generative adversarial networks, image processing, deep learning

## Abstract

With the rapid development of the Internet and information technology (in particular, generative adversarial networks and deep learning), network data are exploding. Due to the misuse of technology and inadequate supervision, deep-network-generated face images flood the network, and the forged image is called a deepfake. Those realistic faked images launched a serious challenge to the human eye and the automatic identification system, resulting in many legal, ethical, and social issues. For the needs of network information security, deep-network-generated face image identification based on different color spaces is proposed. Due to the extremely realistic effect of deepfake images, it is difficult to achieve high accuracy with ordinary methods for neural networks, so we used the image processing method here. First, by analyzing the differences in different color space components in the deep learning network model for face sensitivity, a combination of color space components that can effectively improve the discrimination rate of the deep learning network model is given. Second, to further improve the discriminative performance of the model, a channel attention mechanism was added at the shallow level of the model to further focus on the features contributing to the model. The experimental results show that this scheme achieved better accuracy in the same face generation model and in different face generation models than the two compared methods, and its accuracy reached up to 99.10% in the same face generation model. Meanwhile, the accuracy of this model only decreased to 98.71% when coping with a JPEG compression factor of 100, which shows that this model is robust.

## 1. Introduction

In recent years, with the development of the artificial intelligence technology represented by deep learning, artificial intelligence synthesis techniques have made significant progress in the field of automatic content generation. In the previous image generation field, image generation was usually accomplished with variable autoencoders (VAEs) [1] or autoregressive models [2], but since GAN models have made a big splash in the generation field, variable autoencoders and autoregressive models usually produce poor-quality images compared to GAN, and such generation models limit their applications for generating images, so most of the state-of-the-art generation models are trained with GAN. Currently, the generative adversarial network (GAN) proposed by Goodfellow [3] has been developed from the original GAN model to more advanced GAN models such as DCGAN [4], ProGAN [5], and StyleGAN [6], and these techniques have been applied in various fields of social life, such as webcasting, film and television creation, artwork design, and so on. However, poor regulation and the misuse of technology have led to the use of various types of generated images and videos with realistic effects, which have spread on the Internet. The emergence of new technologies for the human eye, automatic recognition systems, and the long iteration times of devices that cannot easily identify such artificial intelligence attacks have posed serious challenges to the human eye and automatic recognition systems and raised many legal, ethical, and social issues. Compared to other image and video content generation, the faces generated by deep networks are inherently difficult to recognize by the human eye, which leads to serious potential harm and is most likely to cause a social trust crisis. For example, REGIUM replaced real human face images with those generated by AI technology and raised USD 33,111 in eight days.

## 2. Related Works

It is true that many technology companies and academics are responding to the “generation” problem by developing “countermeasures” technologies. McCloskey and Albright [7] discriminated generated images based on the presence of underexposure or overexposure in real face images, and an AUC value of 0.92 was obtained in the classification of ProGAN and Celeba. Matren et al. [8] used the visual artifacts that appear in the eyes, teeth, and facial contours of the generated face images for recognition, and AUC values of 0.852 and 0.843 were obtained for the ProGAN, Glow, and Celeba classifications. Zhang et al. [9] theoretically analyzed the existence of the spectral replication of artifacts in the frequency domain and trained a classifier based on spectral rather than pixel inputs to achieve the effective detection of generated artifacts. The corresponding experimental results on CycleGAN obtained an average accuracy of 97.2. Mo et al. [10] used filters with residuals to design deep learning network models to achieve 96.3% detection accuracy in the corresponding dataset. Dang et al. [11] captured the features of the generated face images by setting a specific CGFace layer with an accuracy of 90.1% for the corresponding dataset. In addition to feeding images directly into deep learning models, some work has attempted to improve detection performance by incorporating domain-specific knowledge. Nataraj [12] trained a network to detect generated artifacts by extracting the co-occurrence matrix from the pixel domain images in RGB space, and it obtained an accuracy of 93.78% after the images were detected by JPEG compression. In a Hsu [13] paper, a new two-stream network structure was proposed by using the simplified DenseNet [14] network structure. This network allows for pairwise learning. Specifically, pairwise learning is used to solve the problem that deep learning network models cannot effectively identify deep-network-generated images that are not included in the training process. In addition, different depth features are extracted in the proposed network structure, and the experimental results were obtained on DCGAN and Celeba with a classification accuracy of 97.2% and a recall accuracy of 91.6%. In a study by Zhuang [15], as in [13], to solve the problem that in order to solve the deep learning network the model cannot effectively identify the deep-network-generated images that are not included in the training process, pairwise learning was used, and, based on this, triplet-state loss was used to learn the relationship between the deep-network-generated images and real images. They also proposed a new coupled network to extract features of different depths of the target image, and the experimental results were obtained on DCGAN and Celeba, with a classification accuracy of 98.6% and a recall accuracy of 98.6%. In a study by Carvalho [16], deep-network-generated faces were detected by finding the differences between real and deep-network-generated faces in the eyes. Specifically, when the eyes’ specular highlights were removed from both real and deep-network-generated faces, the deep-network-generated face presented more artifacts. The bottleneck features obtained using the processed human eyes were extracted using VGG19 [17] for feature extraction. Finally, the eyes were fed into the SVM classifier to classify the deep-network-generated and real faces, and the experimental results obtained an AUC value of 0.88 on the corresponding HD face data.

In summary, the discrimination methods for generating images from generative adversarial networks can be divided into two types: one is to feature the visual artifact defects (e.g., visual artifacts in the eyes, teeth, and facial contours of the generated face images) that exist in the generated images themselves to finally achieve classification discrimination, and the other is to design a specific deep neural network model to achieve the discrimination of the generated face images. Among the related papers mentioned above, papers [7,8,9,16], belong to the first category, using the information of the generated image itself for feature extraction and as an input to the classifier for classification. Papers [10,11,12,13,15], belong to the category of designing specific deep neural network models that are used to implement the classification of the generated face images. The model used in this paper belongs to the second category, where a new image processing method is utilized and then input to a specific neural network for classification.

In this paper, we propose to use different color space channel recombinations on the basis of the existing neural network model to effectively discriminate the generated face graphics. First, by analyzing the differences in different color space components in the deep learning network model for face sensitivity, a combination of color space components that can effectively improve the discrimination rate of the deep learning network model is given. Second, considering the wide application of attention mechanisms in image processing, natural language processing, and speech recognition in recent years, we introduced a channel attention mechanism to the model [18]. When the model has the attention mechanism module at the appropriate location, it enables the model to effectively extract the distinguishable features of real and generated face images. The results of this experiment show that this proposed scheme can effectively solve the recognition problem of face images generated by deep networks. The classification accuracy reached 99.10% on the relevant dataset, and the model possesses good robustness.

## 3. Classification of Neural Network and Attention Mechanism Selection

### 3.1. Classification of Neural Network Selection

Among many mainstream deep learning network models, Xception [19] was chosen as the deep learning network model in this paper.

First, in the analysis literature of mainstream deep learning network models, in the paper by Blanco and Simone [20], the accuracy of Xception belonged to the first echelon and is a lightweight deep learning network model. Second, in the paper on style migration identification using generative adversarial networks by Marra [21], Xception was effective in detecting photos that were not propagated by network compression, and finally, when the test dataset was compressed by the JPEG algorithm, the classification accuracy of the model did not show substantial degradation.

**Figure 1:**
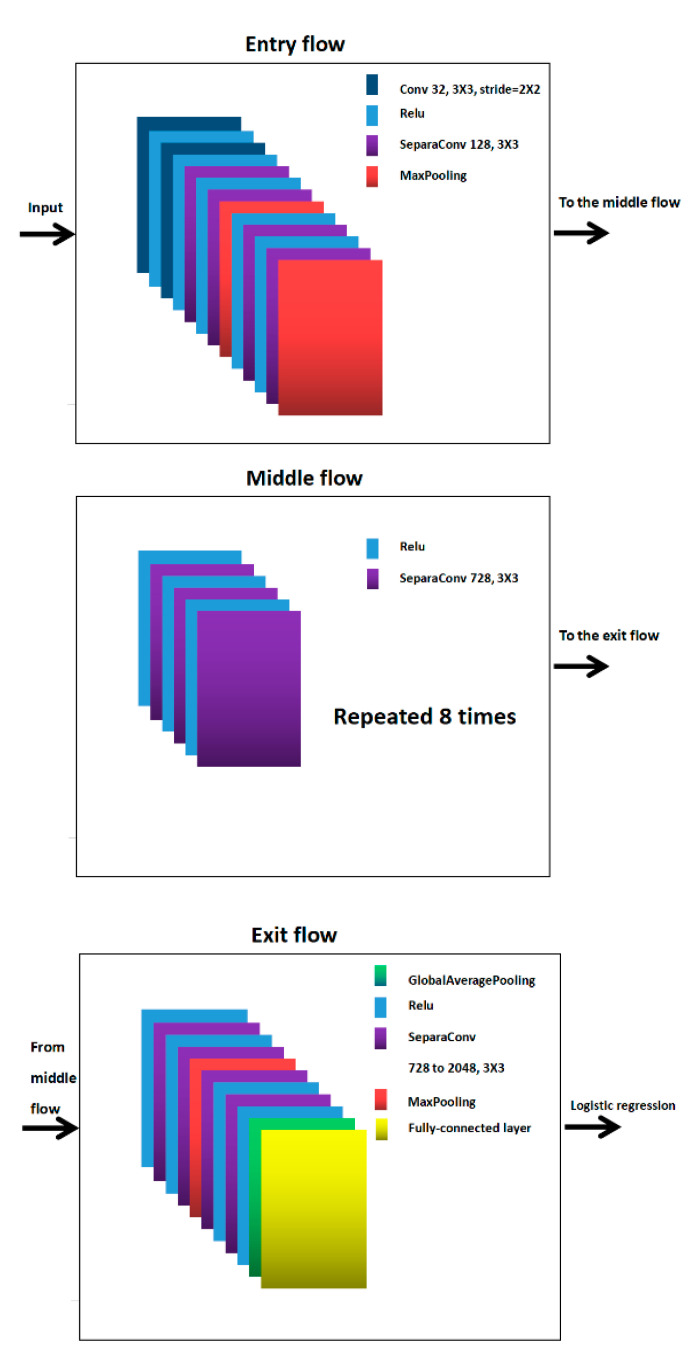
The Xception deep learning network model is divided into three parts: entry flow, middle flow, and exit flow.

The Xception deep learning network model is divided into three parts: entry flow, middle flow, and exit flow. The convolutional kernel size of each layer of the convolutional layer of the entry flow is 3 × 3, and the number of convolutional layer channels gradually increases from 3 to 728. The size of the convolution kernel of each layer of the middle flow is 3 × 3, the number of channels is all 728, and the data tensor is repeated eight times in the intermediate stream. The size of the convolution kernel of the exit flow is 3 × 3, and the number of channels is increased from 728 to 2048 for the final full concatenation. There are residual operations between each convolutional layer. In the structure of Xception, Conv denotes the ordinary convolution operation, SeparaConv is the depth separable convolution (the number of parameters and the cost of operation are relatively low compared to the conventional convolution operation), ReLU is the activation function, MaxPooling is the maximum pooling layer, and GlobalAveragePooling is the global average pooling layer. The simplified structure of Xception is shown in Figure 1.

### 3.2. Attention Mechanism Selection

**Figure 2:**
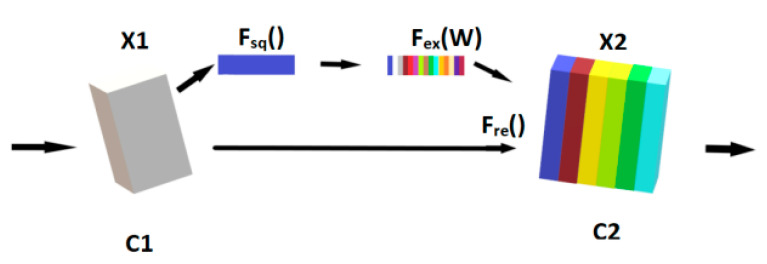
The channel attention module.

Humans have a visual field interaction bottleneck because the brain has a limited processing speed. The human eye does not interact with everything in the visual field; it selects the objects it wants to interact with by way of attention. Therefore, humans can respond quickly to the object of attention despite their limited processing speed. The embodiment of attentional mechanisms in neural learning networks is a framework that is not itself a specific network model. The attention mechanisms are very flexible, such as the common channel attention mechanism module SENet block, and the space attention module CBAM block [22]. In this paper, the channel attention module was chosen, and its structure is shown in Figure 2.

The channel attention operation is performed by squeezing the X1 at F_sq_() with the given number of channels as C1. The global information is generated by performing operations on each channel. Next, the channel activation operation is performed at F_ex_(w), and the weight assignment of each channel is performed by the parameter W. Finally, the weights from the previous step are multiplied by the original feature channels in Fre() to achieve a special focus on important features.

## 4. Deep-Network-Generated Face Image Identification Scheme Design

### 4.1. Deep-Network-Generated Face Dataset

The hardware environment required for this experiment was a Dell T7920 graphics workstation desktop with two Xeon Silver 4210R CPUs, 32 GB of running memory, and an RTX3060 GPU.

In this paper, DCGAN, StyleGAN, and ProGAN were chosen as face image generation models. The training data for generating faces were obtained from the open dataset CelebA of the Chinese University of Hong Kong, which contains 202,599 face images with 178 × 218 pixels.

**Figure 3:**
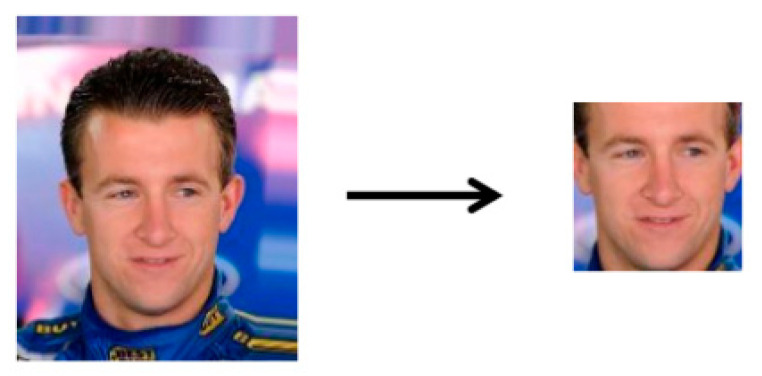
Face interception.

Because the background noise has an impact on the model accuracy, the original CelebA face dataset was used for face interception using the Face_Recognition face recognition library of Python, and the images were intercepted as in Figure 3.

After the face interception, the intercepted face photos of different sizes were reprocessed according to 64 × 64 and 128 × 128 graphic resolutions using the thumbnail method in the Python image package to obtain C64 and C128.

**Figure 4:**
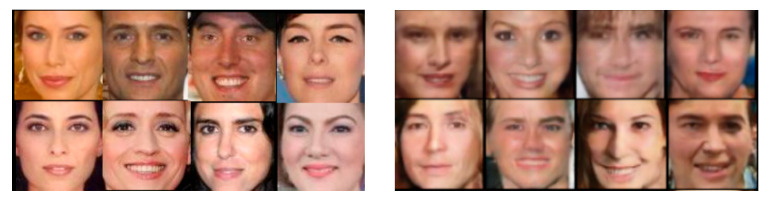
Deep-network-generated face image (left: 128 × 128 and right: 64 × 64).

In order to fairly compare the discrimination rates of face images generated by different GAN models, the resolutions of the face images generated by the three GAN models were set to 128 × 128 and 64 × 64 and are noted as GD128 from DCGAN, GS128 from StyleGAN, GP128 from ProGAN, GD64 from DCGAN, GS64 from StyleGAN, and GP64 from ProGAN. A random sample of 1000 images from the generated faces was evaluated using the face quality evaluation network in Tencent Youtu Open Source [23], and the obtained scores were all above 0.9. Generated faces are shown in Figure 4. The left image of Figure 4 contains 128 × 128 resolution face images produced by StyleGAN, and the right image contains 64 × 64 face images generated by ProGAN.

In this experiment, the mainstream models in the Blanco and Simone paper were used for comparison, including VGG-19 with deeper network depth and a larger number of parameters as well as AlexNet [24] and the residual network ResNet152 [25].

### 4.2. Color Space Channel Processing

An image of a real human face was converted by a camera into an image of electronic data using a light-sensitive charge-coupled device or a complementary metal oxide semiconductor sensor, and the objective scene was recorded digitally in memory. In contrast, deep networks generate face images by continuously training the target dataset with a single model and letting the model generate data with the same distribution as the given target dataset.

**Figure 5:**
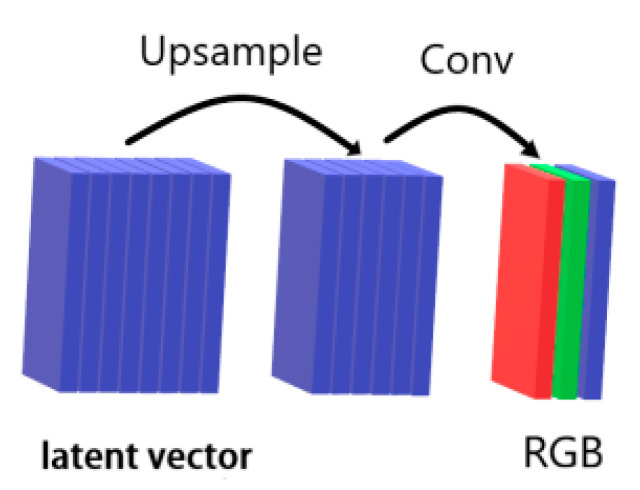
Image generation process.

In the generator of the generative adversarial network, the latter layers convert multiple ‘latent vectors’ into a tensor with three channels, where the three channels represent the R, G, and B channels of the generated image. The channel mapping is shown in Figure 5.

During the image generation process, the generative adversarial network introduces coherence features into the generated images. In contrast to real faces, the color channels of real face images are decomposed and digitized from the real world. This means that real pixels should be intrinsically associated in a different way rather than feature-mapped as in the generated image. In Li’s [26] paper, the HSV and YCbCr color space channels are selected instead of RGB color space channels for the feature training SVM classifier to achieve real face and deep-network-generated face image discrimination.

Before an image was input to the deep learning network model, we processed both the CelebA face dataset and the generated faces in color space, RGB to HSV, and YCbCr color space conversion using R, G, and B components according to the following formulas:

RGB to HSV formula conversion:

(1) V=max(R,G,B)

(2) S=(V−min(R,G,B))×255/V

(3) $$H=\left\{\begin{array}{cc} (G-B)\times 60/S & V=R\\ 180+(B-R)\times 60/S & V=G\\ 240+(R-G)\times 60/S & V=B \end{array}\right.$$

If *H* < 0, H=H+360.

RGB to YCbCr formula conversion:

(4) Y=0.257×R+0.564×G+0.098×B+16

(5) Cb=−0.148×R−0.291×G+0.439×B+128

(6) Cr=0.439×R−0.368×G−0.071×B+128

**Figure 6:**
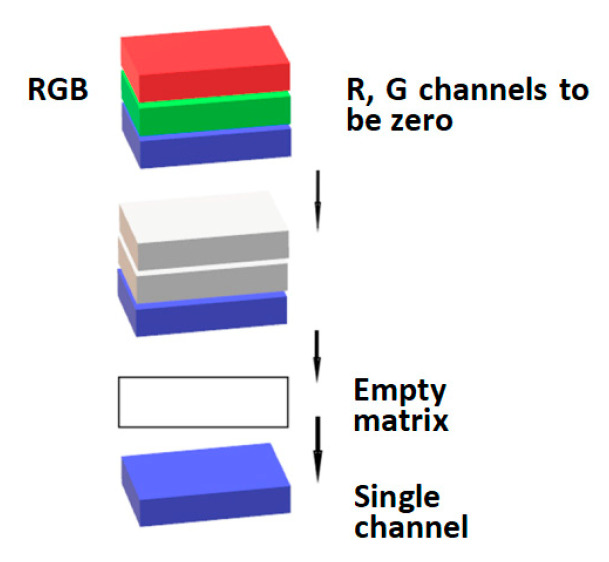
Single-channel acquisition process.

In the extraction of single-channel data after transforming the color space, the two remaining channels needed to be zeroed first (to obtain the H channel in HSV requires the S and V channels to be zeroed). The processed channels were put into an empty matrix prepared in advance, and the single-channel component was obtained. The single-channel extraction is shown in Figure 6.

After the single-channel acquisition of different color spaces, a three-channel image with the same width and height as the original image was created, and then the three-channel reassignment was performed. After that, different color space channels could be fused as required.

### 4.3. Xception Model Optimization

In order to speed up the convergence of the deep learning network model, a pixel value normalization operation was applied to normalize the original channel color value range from [0, 255] to [–1, 1].

This experiment was a binary classification model, so the parameters of this deep learning network were updated using the minimization cross-entropy function. For any output, the binary classification cross-entropy loss function was defined as:

(7) $$L=-\frac{1}{n}\sum_{i=1}^{n}\left[y_{i}\times \ln(p_{i})+(1-y_{i})\times \ln(1-p_{i})\right]$$

In the above equation, *i* is the sample; *y_i_* is the label of *I*; when *y_i_* is 1, it is the real face; when *y_i_* is 0, it is the face generated by the deep learning network; and *p_i_* is the probability that sample *i* is predicted to be a natural face.

### 4.4. Image Channel Recombination

**Figure 7:**
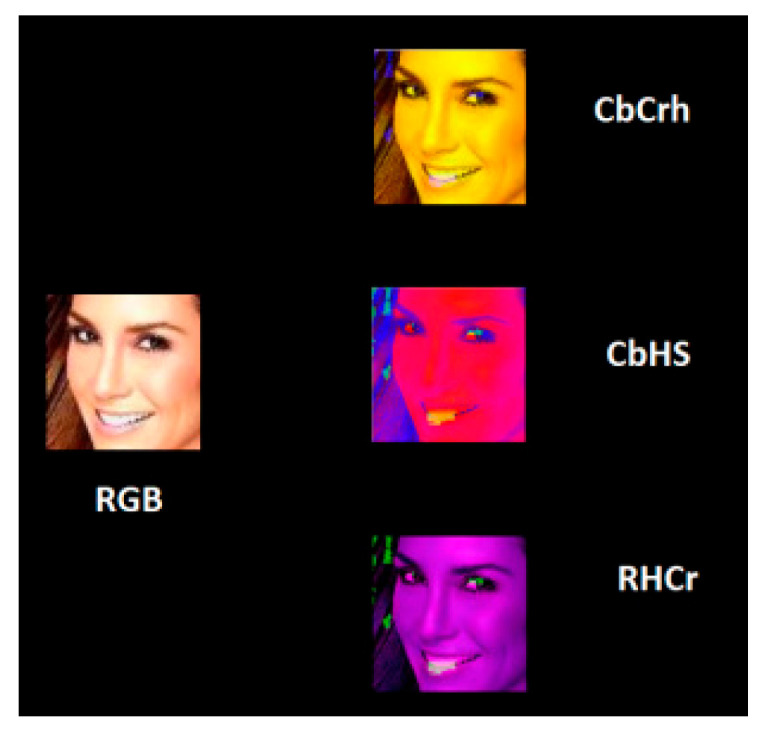
Face image color channel recombination.

After converting RGB to HSV and YCbCr, the channel combinations were selected by R, G, B, H, S, V, Y, Cb, and Cr. The recombined images of the three channels were input into the Xception model to select the top five accuracy combinations of real faces and deep-network-generated faces. Some samples of the transformations are shown in Figure 7.

Before training, the dataset was divided into original images and images with recombined color channels for separate training. A total of 10,000 real face graphics in C128 and 10,000 generated faces in GS128 were randomly selected as the training set, and then 10,000 each of C128 and GS128, which were used for the above data differently, were used as the library test set for testing.

## 5. Results

### 5.1. Deep Learning Network Model Test

A total of 10,000 real face graphics in C128 and 10,000 generated faces in GS128 were randomly selected as the training set, and then the above data were tested using 10,000 images each of different C128 and GS128 faces as the database test set. Different model tests results shown in Table 1.

The above results show that Xception is competent in this task.

### 5.2. Attentional Mechanisms and Image Channel Preprocessing Ablation Experiments

For a deep learning network model, due to the optimal size of the receptive field in one layer of the network, adding modules to expand or reduce the receptive field in the corresponding layer can have side effects. Therefore, different position insertion attention mechanism modules were performed to verify that the best embedding position was obtained.

In order to compare the effect of embedding attention at different locations on the accuracy of the model, the attention mechanism was added to the second layer of the entry flow, the second layer of the middle flow, and the fourth layer of the exit flow of the Xception network. The training dataset was the same as in Section 5.1. The effect is shown in Table 2.

From Table 2, it can be seen that more global information about the face could be noticed when the attention mechanism was at a shallow level of the network. Above, it was concluded that the model accuracy was best when the input stream was embedded in the channel attention mechanism.

The combinations of the three-color spaces with different channels were input to the Xception network without channel attention. The combination with the top five accuracy values was taken, and in addition, the original color space was taken again. The training dataset was the same as in Section 5.1. The obtained results are shown in Table 3.

Table 3 shows that the highest accuracy was obtained by combining the HSV and YCbCr color spaces, unlike the native RGB space, which was not as effective as the HSV and YCbCr color spaces. This is also consistent with the results shown in Li’s paper, in that HSV and YCbCr contributed much more to the model in the color space than the original RGB space.

The results obtained by recombining the image channels as H, S, and Cb and then feeding the images to Xception, which has a channel attention mechanism module. The training dataset was the same as in Section 5.1. The results are shown in the following Table 4.

The above results show that the channel attention method and the image channel recombination method mutually enhanced each other in this ablation experiment.

### 5.3. Comparison of Methods

In this section, we compare the proposed approach with the network model structure in papers [5] (MO) and [6] (Dang).

In total, 10,000 real face graphics in C128 and 10,000 generated faces in GS128 were randomly selected as training sets, and then the above data were tested using different selected images (10,000 in C128 and 10,000 in GS128) as the database test sets. Then, there were GD128, GP128, GD64, GS64, and GP64, each corresponding to C128, C128, C64, C64, and C64 data that were mutually exclusive with the training set, and the number of each was 10,000. The results are shown in Table 5.

**Figure 8:**
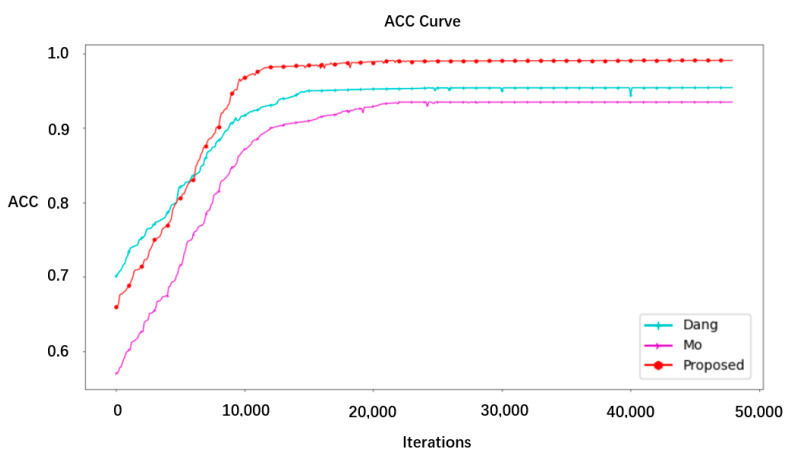
Accuracy curves of the different models.

The accuracy performance results of the three methods on GS128 and C128 are shown in the Figure 8.

**Figure 9:**
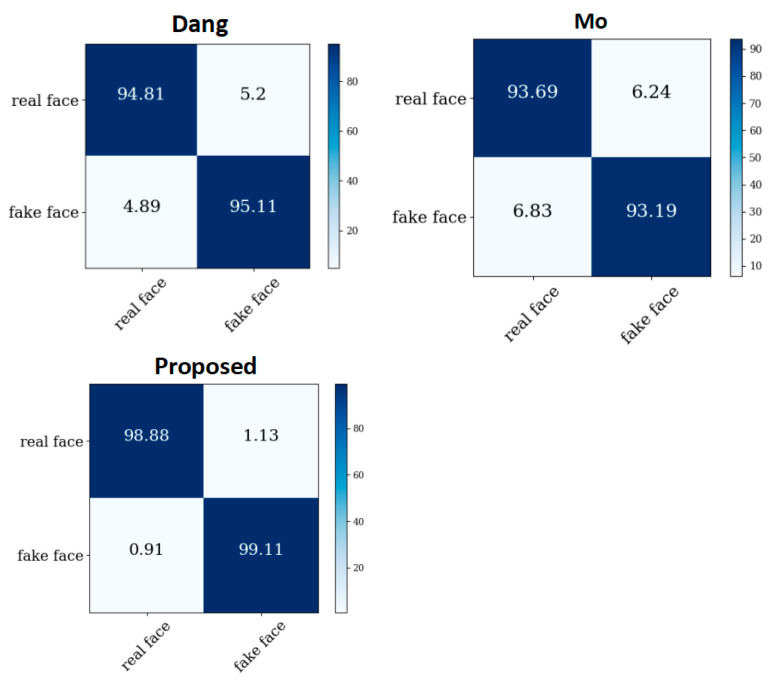
The confusion matrix performances of the three models on GS128 and C128.

The confusion matrix performances of the three models on GS128 and C128 are shown in Figure 9.

From the figure, it can be seen that the method proposed in this paper was superior to the other two schemes in terms of convergence speed and accuracy, and this network is a lightweight network that is easy to deploy industrially.

### 5.4. Robustness Testing

The model is affected by the specific image compression that is performed when false images are uploaded on the Internet, so a test set was generated for a face image compression operation to test the model’s robustness, while the weight training of the deep learning network model was still trained by the uncompressed training set.

JPEG compression is often used for compression during network transmission, and to test the model’s robustness, a test of image compression was performed by quality factors ranging from 70 to 100 with an interval of 10. The training dataset was the same as in Section 5.1. The results are shown in Table 6.

As can be seen from Table 6, the robustness of the present model is excellent.

## 6. Discussion

In this proposed method, we used a new image preprocessing approach to discriminate the faces generated by deep learning networks, in that the pixel synthesis from the deep-network-generated face images themselves is not the same as the real face synthesis approach. Because generative adversarial networks do not represent many details in the generated images as correctly as real images, this also leads to potential factors in the generated images that can be explored. Therefore, it is these potential factors that are different from the real face that we can use to assist our model in classification. In this paper, the potential factor was that the images generated by the generative adversarial network were expressed differently from the real images in different color spaces. In the future, our work should also focus on this aspect: finding the differences between the faces generated by deep learning networks and real faces.

The proposed method of image processing can also be used in other deepfake fields, such as the statistical analysis of spectrograms of channel-recombined images to achieve classification effects. Can this solve the problem of an insufficient number of samples?

Even though our proposed method achieved high classification accuracy in the corresponding DCGAN, ProGAN, and StyleGAN datasets and the model still had good robustness against JPEG compression attacks, we find that the classification accuracy of the model decreased when the training dataset for generating faces was GS128 and the test dataset was not the same model. Its classification accuracy decreased from 99.10% to 95.55% on average for some datasets (99.10% was removed), and it decreased more rapidly with the [5] (MO) and [6] (Dang) methods, corresponding to average decreases of 89.93% and 86.62%. In the future, we will focus on the commonalities between the different generative models, with the initial intention to work on domain migration.

## 7. Conclusions

For the problem of discriminating deep-network-generated faces and real face images, this paper proposes a method based on color space combination. By the different sensitivities of the different color space components of faces, a color space component combination method that can effectively improve the discrimination rate of deep learning network models is given. Accuracy experiments with different mainstream models demonstrated the advantages of Xception in discriminating between deep-network-generated faces and real faces. In addition, the attention mechanism affected the receptive field of the network, leading to a change in the optimal perceptual field and thus reducing the model accuracy. Therefore, in this paper, different depths of the network were assessed to insert attention mechanisms to verify the location where the best embedded attention mechanism was obtained, and it was concluded that the shallow insertion of channel attention in this Xception network model contributed the most to the accuracy rate. Finally, the combination of color components H, S, and Cb together with the attention mechanism obtained an accuracy of 99.10% for the test set, and the proposed scheme outperformed the other schemes in the comparison of different methods.

Although the generated face images were visually indistinguishable by the human eye, the method proposed in this paper could effectively identify that they represented many intrinsic properties possessed by real images (e.g., properties in different color components) that were not properly represented by the generative model. In future work, the generative model will be further explored in the attributes that cannot be correctly expressed.

## Figures and Tables

**Table 1:** Accuracy of each model.

Deep Learning Networks	Accuracy
Xception	94.46%
ResNet152	91.74
VGG-19	92.32
AlexNet	87.95%

**Table 2:** Channel attention test in different positions.

Network Depth	Accuracy Improvement
Entry flow	96.82%
Middle flow	89.42%
Exit flow	91.74%

**Table 3:** Experimental results of different channel combinations (model network without channel attention module).

Channel Recombination	Test Set Accuracy
H, S, Cb	97.32%
H, S, Cr	96.41%
H, Cb, Cr	95.79%
Cb, Cr, H	95.70%
Cb, Cr, S	95.43%
HSV	95.37%
YCbCr	95.32%
RGB	94.46%

**Table 4:** Channel attention combined with image preprocessing.

The Above Two Methods	Test Set Accuracy
H, S, Cb	99.10%

**Table 5:** Comparison of the results of different models.

Model	GS128	GD128	GP128	GD64	GS64	GP64	Average
Mo	93.50%	90.32%	91.73%	86.52%	84.39%	80.21%	87.78%
Dang	95.43%	92.18%	96.37%	90.62%	80.24%	90.24%	90.85%
Proposed	99.10%	98.82%	99.05%	95.78%	90.32%	93.78%	96.14%

**Table 6:** Impact of different JPEG quality factor attacks on the model.

	70	80	90	100
Dang	81.69%	90.34%	91.35%	93.54%
Mo	76.42%	79.95%	85.23%	90.21%
Proposed	85.38%	93.79%	97.64%	98.71%

## Data Availability

Not applicable.
